# Genetically predicted 1091 blood metabolites and 309 metabolite ratios in relation to risk of type 2 diabetes: a Mendelian randomization study

**DOI:** 10.3389/fgene.2024.1356696

**Published:** 2024-07-10

**Authors:** Jixin Li, Wenru Wang, Fengzhao Liu, Linjie Qiu, Yan Ren, Meijie Li, Wenjie Li, Feng Gao, Jin Zhang

**Affiliations:** ^1^ Xiyuan Hospital of the China Academy of Chinese Medical Sciences, Beijing, China; ^2^ First Clinical Medical College, Shandong University of Traditional Chinese Medicine, Jinan, China

**Keywords:** T2DM, blood metabolites, metabolite ratios, Mendelian randomization, genomewide association study

## Abstract

**Background:**

Metabolic dysregulation represents a defining characteristic of Type 2 diabetes (T2DM). Nevertheless, there remains an absence of substantial evidence establishing a direct causal link between circulating blood metabolites and the promotion or prevention of T2DM. In addressing this gap, we employed Mendelian randomization (MR) analysis to investigate the potential causal association between 1,091 blood metabolites, 309 metabolite ratios, and the occurrence of T2DM.

**Methods:**

Data encompassing single-nucleotide polymorphisms (SNPs) for 1,091 blood metabolites and 309 metabolite ratios were extracted from a Canadian Genome-wide association study (GWAS) involving 8,299 participants. To evaluate the causal link between these metabolites and Type 2 diabetes (T2DM), multiple methods including Inverse Variance Weighted (IVW), Weighted Median, MR Egger, Weighted Mode, and Simple Mode were employed. *p*-values underwent correction utilizing False Discovery Rates (FDR). Sensitivity analyses incorporated Cochran’s Q test, MR-Egger intercept test, MR-PRESSO, Steiger test, leave-one-out analysis, and single SNP analysis. The causal effects were visualized via Circos plot, forest plot, and scatter plot. Furthermore, for noteworthy, an independent T2DM GWAS dataset (GCST006867) was utilized for replication analysis. Metabolic pathway analysis of closely correlated metabolites was conducted using MetaboAnalyst 5.0.

**Results:**

The IVW analysis method utilized in this study revealed 88 blood metabolites and 37 metabolite ratios demonstrating a significant causal relationship with T2DM (*p* < 0.05). Notably, strong causal associations with T2DM were observed for specific metabolites: 1-linoleoyl-GPE (18:2) (IVW: OR:0.930, 95% CI: 0.899–0.962, *p* = 2.16 × 10^−5^), 1,2-dilinoleoyl-GPE (18:2/18:2) (IVW: OR:0.942, 95% CI: 0.917–0.968, *p* = 1.64 × 10^−5^), Mannose (IVW: OR:1.133, 95% CI: 1.072–1.197, *p* = 1.02 × 10^−5^), X-21829 (IVW: OR:1.036, 95% CI: 1.036–1.122, *p* = 9.44 × 10^−5^), and Phosphate to mannose ratio (IVW: OR:0.870, 95% CI: 0.818–0.926, *p* = 1.29 × 10^−5^, FDR = 0.008). Additionally, metabolic pathway analysis highlighted six significant pathways associated with T2DM development: Valine, leucine and isoleucine biosynthesis, Phenylalanine metabolism, Glycerophospholipid metabolism, Alpha-Linolenic acid metabolism, Sphingolipid metabolism, and Alanine, aspartate, and glutamate metabolism.

**Conclusion:**

This study identifies both protective and risk-associated metabolites that play a causal role in the development of T2DM. By integrating genomics and metabolomics, it presents novel insights into the pathogenesis of T2DM. These findings hold potential implications for early screening, preventive measures, and treatment strategies for T2DM.

## 1 Introduction

New data released by the International Diabetes Federation (IDF) in 2021 indicates a global prevalence of 537 million adults diagnosed with diabetes mellitus (DM). This represents a 16% increase from the 2019 forecast. Projections suggest a further rise to 643 million by 2030 and a staggering 783 million by 2045 (Ogurtsova et al., 2022). Type 2 diabetes (T2DM) manifests as a metabolic disorder typified by both insulin resistance and insufficient insulin secretion (Keller-Pinter et al., 2023). Insulin resistance represents an initial stage in the progression of T2DM. It affects approximately 40% of young adults in the United States (Parcha et al., 2022). The World Health Organization predicts that the global number of T2DM patients will double, reaching 350 million by 2030 (Collins et al., 2011). Type 2 diabetes mellitus (T2DM) is associated with complications like renal disease, coronary heart disease, peripheral vascular disease, and other systemic issues, causing a mortality rate twice as high as that of the healthy population as the disease progresses (Mulnier et al., 2006). In recent years, T2DM has shown a trend towards affecting individuals at a younger age, exhibiting prolonged disease duration, multiple complications, and significant risks (Magliano et al., 2020). It imposes a substantial economic burden globally and stands as a critical public health concern. Identifying risk factors for T2DM and proactively managing high-risk individuals holds immense significance in preventing and treating this condition.

Insulin resistance stands prominently as a pivotal factor in the pathogenesis of T2DM, driven not solely by the compromised transmission of the insulin signaling pathway but also entailing a myriad of intricate metabolic determinants (Yang et al., 2018). For instance, saturated fatty acids exhibit the potential to impair insulin sensitivity in murine models (Holland et al., 2011). Conversely, judicious intake of unsaturated fatty acids holds promise in ameliorating insulin sensitivity and mitigating the risk of T2DM (Imamura et al., 2016). Moreover, more than 4 decades ago, it was discerned that heightened serum levels of certain amino acids strongly correlate with obesity and insulin resistance (Felig et al., 1969). Metabolites serve as the foundation of biological expression, providing crucial insights into metabolic processes and disease pathogenesis (Bauermeister et al., 2022; Johnson et al., 2016). Metabolomics allows for the exploration of metabolic pathways or networks through qualitative and quantitative analysis of metabolites, unveiling the metabolic and reaction mechanisms of various diseases, drugs, and chemicals across different organisms (Johnson et al., 2016). Metabolomics enables a thorough characterization of serum metabolite alterations in patients with T2DM both pre- and post-onset, as well as following treatment initiation. Lee (Newgard et al., 2009), through untargeted metabolomics, identified the plasma metabolite Branched Chain Amino Acid (BCAA), showing a negative correlation with insulin sensitivity and insulin metabolic clearance. Similarly, Marina (Mora-Ortiz et al., 2022) identified 12 metabolites predictive of T2DM remission over a 5-year dietary intervention in 190 T2DM patients. This highlights how metabolomics technology facilitates understanding the mechanisms underlying diabetes development. While numerous studies have explored the correlation between metabolites and T2DM(Bloomgarden, 2018; Long et al., 2020; Mora-Ortiz et al., 2022; Newgard et al., 2009; Palmer et al., 2015; Curtin and Schulz, 1998; Wang et al., 2017), the coverage of metabolites remains incomplete. None have comprehensively accounted for the influence of confounding factors, potentially leading to biased results.

Genomics and metabolomics are closely intertwined. Their integration enables the identification of key mechanisms underlying the development of T2DM and facilitates the exploration of disease markers and drug targets. Mendelian randomization (MR) serves as an epidemiological and genetically grounded method, exploring causal links between exposures and outcomes. Adhering to Mendel’s second law, MR studies are not influenced by confounding factors due to the random assignment of alleles during gamete formation. This study integrates genomics and metabolomics, leveraging the latest comprehensive genome-wide association study (GWAS) on blood metabolites (Chen et al., 2023). It employs Mendelian randomization (MR) analysis to investigate the causal associations of 1,091 blood metabolites and 309 metabolite ratios with T2DM. The objective is to comprehensively dissect the pathogenesis of T2DM and its metabolic pathways, thereby providing insights into prediction, diagnosis, and treatment.

## 2 Materials and methods

### 2.1 Reporting guidelines

This study was reported in strict adherence to STROBE-MR guidelines (Skrivankova et al., 2021).

### 2.2 Research design

**FIGURE 1:**
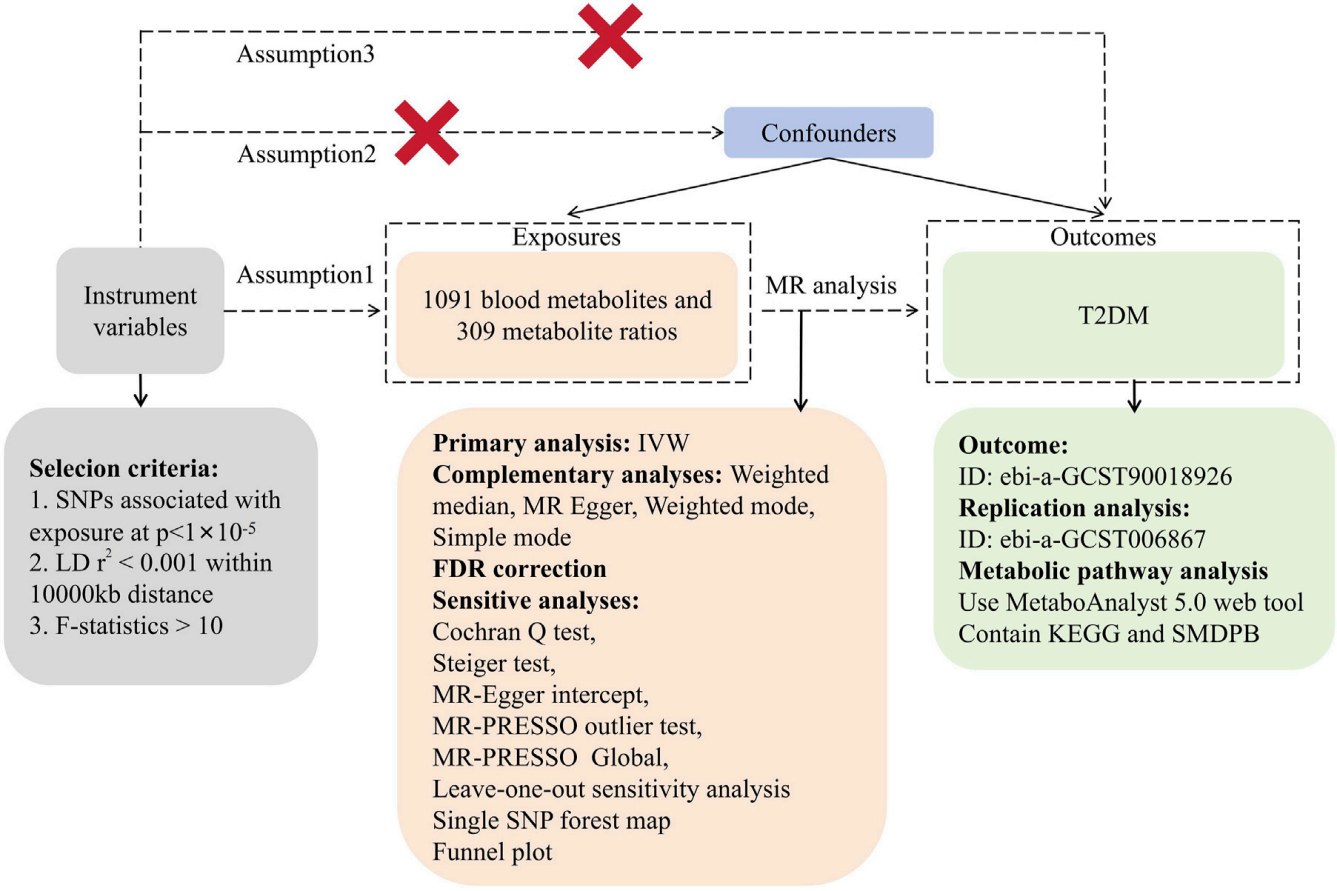
Research flowchart.

In this investigation, we utilized 1,091 blood metabolites and 309 metabolite ratios as ‘exposures’ while considering T2DM as the outcome. Instrumental variables (IVs) were meticulously screened for MR analysis. The study’s consistency underwent evaluation using the Cochran Q test. Sensitivity analyses encompassed horizontal multiplicity analysis and a ‘leave-one-out’ approach, reinforcing the reliability of our findings. Mendelian randomization (MR) studies require adherence to three fundamental assumptions: (1) establishing a robust correlation between IVs and exposure, (2) ensuring IVs’ independence from any confounding factors associated with exposure and outcome, and (3) affirming that IVs solely impact outcomes through exposure pathways. In our investigation, MR analysis was employed to ascertain the causal relationship between 1,091 blood metabolites, 309 metabolite ratios, and T2DM. Figure 1 illustrates the study’s workflow (Figure 1).

### 2.3 Data sources

The instrumental variables for this study were sourced from Chen’s Canadian Longitudinal Study of Aging (CLSA), encompassing a sample size of 8,299 (Chen et al., 2023). CLSA recruited 51,338 Canadians aged 45–85 years, incorporating concurrent statistical data on their physiology, lifestyle, and economic status. A metabolomics investigation was conducted on 8,299 unrelated subjects within the CLSA cohort, measuring their plasma metabolites and conducting genome-wide association analyses. This study represents the most recent and comprehensive genome-wide association study of metabolites, encompassing data on 1,091 plasma metabolites and 309 plasma metabolite ratios. For further details on the GWAS, please refer to the original publication (Chen et al., 2023). The instrumental variables for T2DM were acquired through the IEU Open GWAS database (ID: ebi-a-GCST90018926). The dataset utilized in this study originated from Sakaue’s meta-analysis, incorporating data from the United Kingdom Biobank and FinnGen (Sakaue et al., 2021). GWAS data for T2DM were extracted from this meta-analysis, which included a total of 24,167,560 SNPs identified across 4,90089 individuals of European ancestry. To the best of our knowledge, there is no sample overlap between exposures and outcomes in the current study, rendering the data relatively reliable.

### 2.4 Screening of instrumental variables

To identify qualified single-nucleotide polymorphisms (SNPs), we established a screening threshold. In this study, we adopted a relaxed association threshold of *p* < 1 × 10^−5^ (Xiao et al., 2022) to identify SNPs strongly associated with 1,400 exposures. Additionally, to mitigate the influence of linkage disequilibrium (LD), we implemented criteria of *r* ^2^ < 0.001 and a kilobase pair (kb) value of 10,000. To diminish the impact of weak instrumental variables (IVs) on the study’s outcomes, we assessed completed SNPs using the F-statistic (Palmer et al., 2012). A threshold of F-statistic >10 indicated a low likelihood of the IV being weak (von Zglinicki, 2002). Furthermore, to mitigate the influence of additional potential confounders on the study outcome variables, we employed the Phenotype Scanner V2.0 database (Kamat et al., 2019) to exclude SNPs correlated with factors such as smoking, alcohol consumption, physical activity, and education level.

### 2.5 Statistical analysis

We utilized the TwoSampleMR software package of R4.2.3 to analyze 1,091 blood metabolites and 309 metabolite ratios in relation to T2DM through MR. This study employed five commonly used analytical methods, namely, Inverse variance weighted (IVW), Weighted median, MR Egger, Weighted mode, and Simple mode, to determine their causal associations with T2DM.The IVW method, the primary approach in MR analysis, assumes the validity of each SNP, enabling a reliable assessment of exposure’s causal effect on the outcome (Hartwig et al., 2017). MR Egger regression analysis accommodates potential pleiotropy or a substantial number of invalid instrumental variables for causal inference (Bowden et al., 2015). The Weighted median approach is utilized when at least 50% of valid instrumental variables are presumed to be present (Slob and Burgess, 2020). Weighted mode and Simple mode are alternate methods that relax assumptions, albeit with lower test efficacy compared to the previous three methods (Hartwig et al., 2017). These methods serve as supplementary tools for MR analysis. Additionally, to address multiple comparisons, False Discovery Rates (FDR) proposed by Benjamini and Hochberg were applied to correct *p*-values in the IVW results (Curtin and Schulz, 1998). A significance threshold of *p* < 0.05 after FDR correction was utilized.

### 2.6 Sensitivity analysis

To ensure result quality, a comprehensive sensitivity analysis was conducted using TwoSampleMR and MR-PRESSO software packages within R 4.2.3. Cochran’s Q test assessed SNP heterogeneity (Greco et al., 2015). The MR-Egger intercept test and MR-PRESSO Global examined pleiotropy. Horizontal pleiotropy suggests a non-causal association pathway between IVs and outcomes, potentially causing false positives (Hemani et al., 2018). MR-PRESSO identified and excluded significant outliers, if present, followed by a reiteration of the MR analysis. Additionally, the Steiger test was applied to mitigate bias arising from reverse causality (Hemani et al., 2017). Individual SNP effect analyses and leave-one-out sensitivity analyses were performed to identify SNPs susceptible to significant heterogeneity.

### 2.7 Replication analysis

To further validate the associations identified in the primary analysis, we conducted replication analyses of metabolites and metabolite ratios initially found to exhibit significant causal associations (FDR <0.05) using independent GWAS data for blood metabolites. For this purpose, we utilized the IEU Open GWAS database (ID: ebi-a-GCST006867), which provided the largest sample size GWAS data available for T2DM, comprising 659,316 individuals of European ancestry. This dataset, derived from a meta-analysis conducted by Xue et al. (2018), incorporated raw data primarily sourced from datasets such as the DIAbetes Genetics Replication And Meta-analysis (DIAGRAM) and Genetic Epidemiology Research on Adult Health and Aging (GERA), totaling 62,892 T2DM cases and 596,424 controls of European ancestry, and encompassing over five million genetic variants. For additional details regarding the GWAS, please refer to the original publication (Xue et al., 2018). The Mendelian randomization (MR) analysis methods employed in the replication analyses were consistent with those utilized in the main analyses and comprised the IVW, weighted median, MR Egger, weighted mode, and simple mode approaches, with the IVW results serving as the primary outcomes. Furthermore, a comprehensive sensitivity analysis was conducted to assess the robustness of the findings. Replication analyses were conducted to validate the reliability of our results.

### 2.8 Metabolic pathway analysis

Metabolic pathway analysis was conducted via MetaboAnalyst 5.0 for the metabolites exhibiting significant causal associations with T2DM identified through the IVW method in this study (https://www.metaboanalyst.ca/) (Chong et al., 2018). MetaboAnalyst 5.0 serves as a comprehensive web-based data analysis tool designed to aid users in metabolomics data analysis and visualization. Utilizing this platform enables the identification of potential metabolite pathways associated with the underlying biological mechanisms of T2DM.

## 3 Results

### 3.1 Instrumental variables

After a meticulous series of screening steps, a final set of 1,091 blood metabolites and 309 metabolite ratios were obtained, featuring a range of 12–93 IVs. Among the 1,091 blood metabolites, IVs varied from 12 to 93, with X-15523 yielding the highest number of IVs and X-12462 the lowest. Similarly, the 309 metabolite ratios exhibited IVs ranging from 13 to 39, with the glutamine to alanine ratio presenting the highest number and the adenosine 5′-diphosphate to uridine ratio the fewest IVs. The F-statistic of the SNPs analyzed in this study ranged from 19.50 to 5,308.35, indicating a minimal likelihood of weak instrumental variables. These findings support the validity of all IVs for conducting Mendelian randomization analyses involving the 1,091 blood metabolites and 309 metabolite ratios (Supplementary Table S1).

### 3.2 Causal association of 1,091 blood metabolites and 309 metabolite ratios on T2DM

**FIGURE 2:**
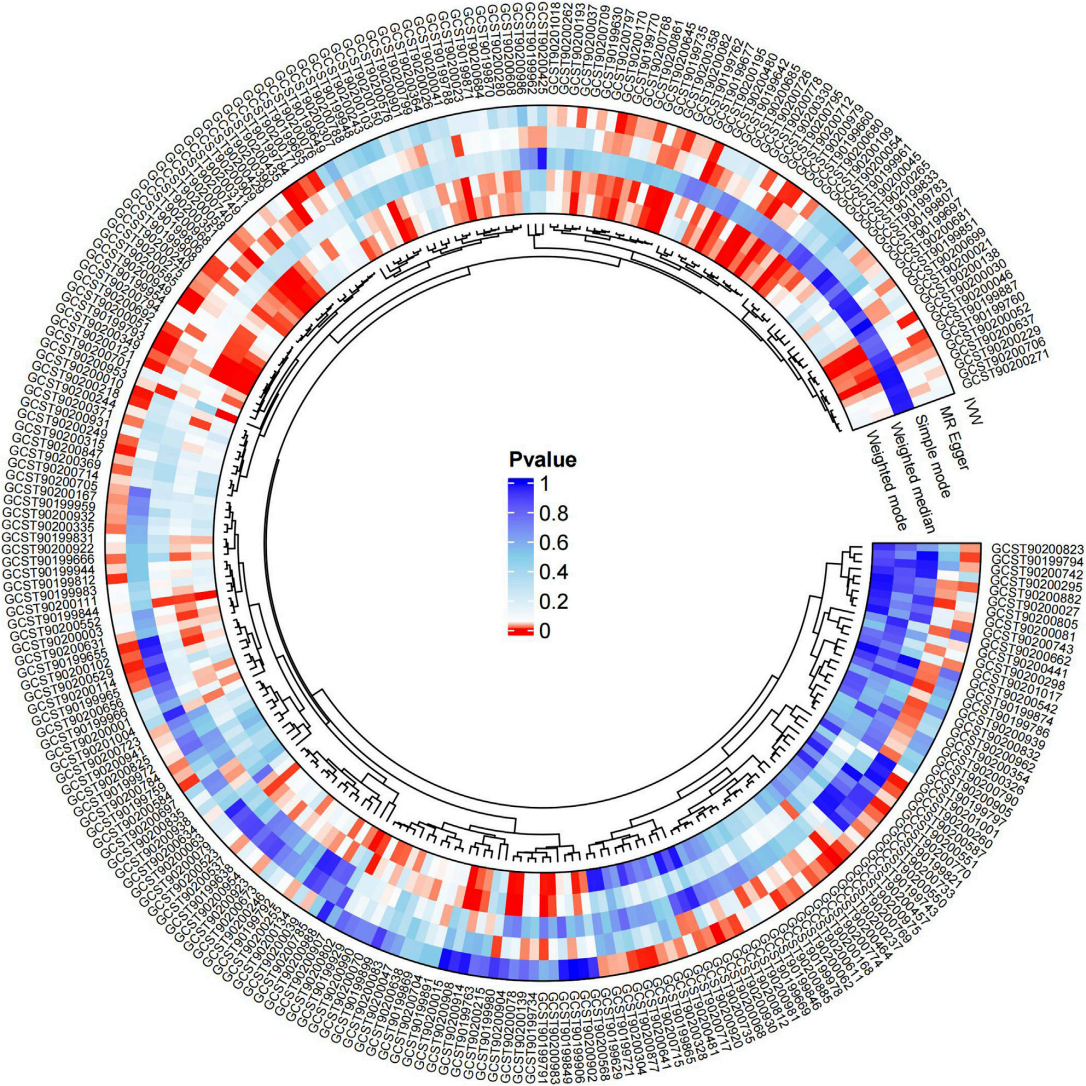
Circos plot of MR analysis results for 185 blood metabolites and 70 metabolite ratios.

**FIGURE 3:**
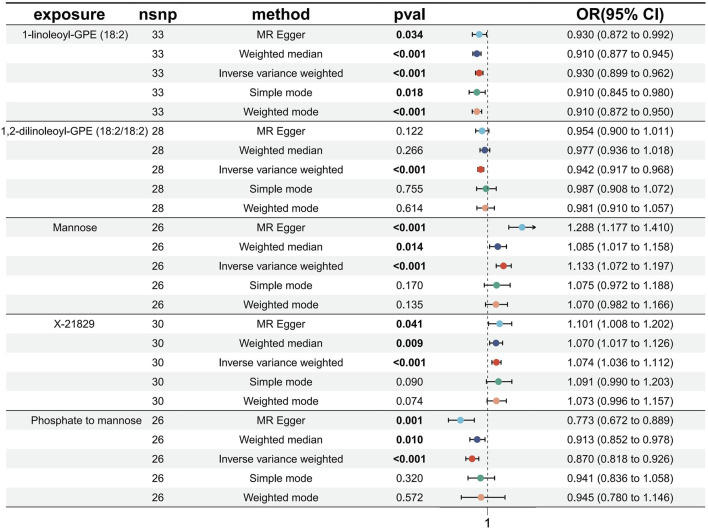
Forest plot of FDR-corrected significant results.

**FIGURE 4:**
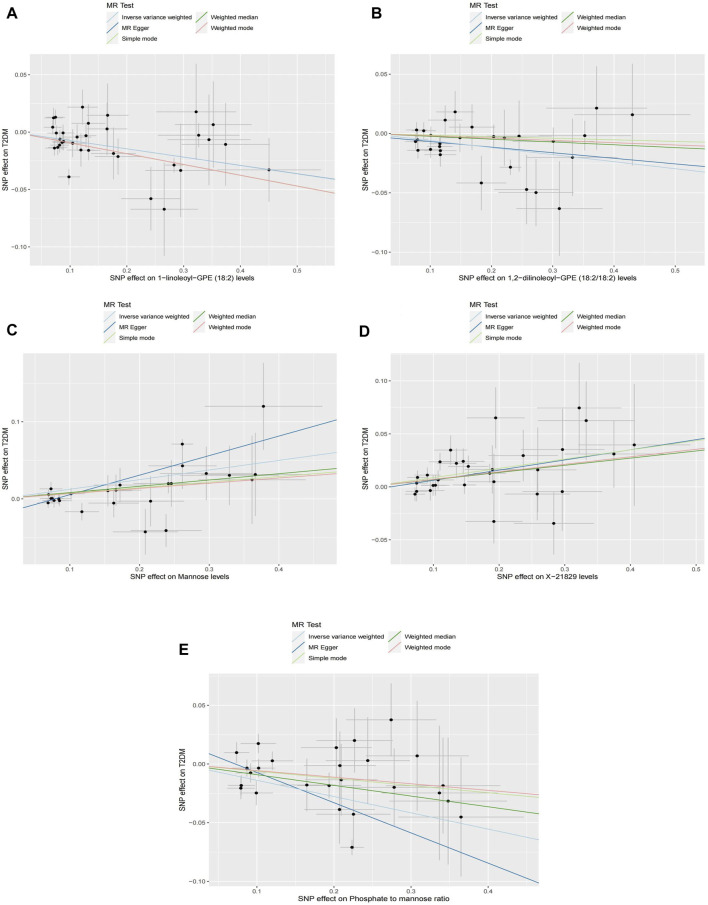
Scatterplot of causal associations between core metabolites and Phosphate to mannose on T2DM Note: **(A)** 1-linoleoyl-GPE (18:2) on T2DM. **(B)** 1,2-dilinoleoyl-GPE (18:2/18:2) on T2DM. **(C)** Mannose on T2DM. **(D)** X-21829 on T2DM. **(E)** Phosphate to mannose on T2DM.

Causal associations of 1,091 blood metabolites and 309 metabolite ratios for T2DM were determined by 5 MR analysis methods (IVW, Weighted median, MR Egger, Weighted mode and Simple mode). We identified a total of 185 blood metabolites and 70 metabolite ratios (*p* < 0.05 for the presence of at least 1 MR analysis method) with a significant causal association with T2DM (Supplementary Table S2). Visualize this result with a circos plot (Figure 2). The figure comprises 1,275 color blocks, each representing an individual MR analysis result for one of the exposures. Different colors denote various *p*-values, with shades closer to dark blue indicating larger *p*-values and those closer to red indicating smaller *p*-values. The IVW analysis identified 88 blood metabolites and 37 metabolite ratios as having a significant causal relationship with T2DM (*p* < 0.05) (Supplementary Table S3). It contains 74 known metabolites. After correction based on the FDR method, a total of three known blood metabolites, one unknown blood metabolite, and one metabolite ratios were identified as having a significant causal relationship with T2DM (*p* < 0.05) (Supplementary Table S4). They are respectively: 1-linoleoyl-GPE (18:2) (IVW: OR:0.930, 95%CI: 0.899–0.962, *p* = 2.16 × 10^−5^, FDR = 0.008), 1,2-dilinoleoyl-GPE (18:2/18:2) (IVW: OR:0.942, 95%CI: 0.917–0.968, *p* = 1.64 × 10^−5^, FDR = 0.008), Mannose (IVW: OR:1.133, 95%CI: 1.072–1.197, *p* = 1.02 × 10^−5^, FDR = 0.014), X-21829 (IVW: OR:1.036, 95%CI: 1.036–1.122, *p* = 9.44 × 10^−5^, FDR = 0.026), Phosphate to mannose ratio (IVW: OR:0.870, 95%CI: 0.818–0.926, *p* = 1.29 × 10^−5^, FDR = 0.008). This result is visualized through a forest plot (Figure 3). The bolded *p*-values in the figure indicate statistical significance, while the five colored nodes represent five distinct MR analysis methods. Among them 1-linoleoyl-GPE (18:2), 1,2-dilinoleoyl-GPE (18:2/18:2) and Phosphate to mannose ratio were protective factors for T2DM and X-21829 and Mannose were risk factors for T2DM. This result is further visualized by means of a scatterplot (Figure 4). The graph displays five distinct colored line segments, each corresponding to a different method of MR analysis. The slope of the lines indicates the direction of the causal association, whether positive or negative.

### 3.3 Sensitive analysis

We conducted a sensitivity analysis to evaluate the stability of the five exposures that exhibited IVW results consistent with FDR<0.05 correction. The Cochran Q test indicated no significant heterogeneity between the SNPs of the IVW method and the MR-Egger method (*p* > 0.05) (Supplementary Table S5). Both the MR-Egger intercept test and the MR-PRESSO Global test results suggested the absence of horizontal pleiotropy in our study (*p* > 0.05) (Supplementary Table S6). Additionally, the MR-PRESSO outlier test did not detect significant outlier SNPs. Our leave-one-out sensitivity analyses and the MR analyses of individual SNPs (Supplementary Figures S1–S10) demonstrated the robustness of our MR analyses, with no instances of individual SNPs significantly influencing the outcomes. Moreover, the Steiger test for directionality resulted in TRUE (*p* < 0.05) in our study. Notably, the funnel plot illustrated a generally symmetrical distribution of IVW results in this study without notable bias (Supplementary Figures S11–S15). These findings collectively contribute to the increased reliability of the results obtained in this study.

### 3.4 Replication analysis

**FIGURE 5:**
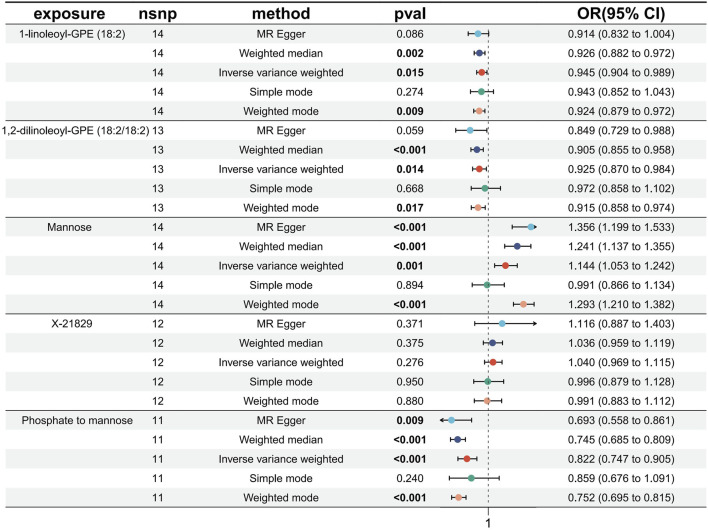
Forest plot of Replication analysis results.

To bolster the credibility of our study’s findings, we conducted a replication analysis on the five exposures meeting the FDR<0.05 correction criteria using an alternative GWAS dataset for T2DM (Xue et al., 2018). Encouragingly, our observations within this GWAS outcome data aligned with similar trends for these five exposures. However, the unknown metabolite X-21829 did not exhibit a significant difference concerning T2DM. Among these, three metabolites: 1-linoleoyl-GPE (18:2) (IVW: OR:0.945, 95%CI: 0.904–0.989, *p* = 0.015), 1,2-dilinoleoyl-GPE (18:2/18:2) (IVW: OR:0.925, 95%CI: 0.870–0.984, *p* = 0.014), Mannose (IVW: OR:1.143, 95%CI: 1.053–1.241, *p* = 0.001), and Phosphate to mannose ratio (IVW: OR:0.822, 95%CI: 0.747–0.905, *p* = 6.64 × 10^−5^) maintained their significant causal associations with T2DM (Figure 5). The bolded *p*-values in the figure indicate statistical significance, while the five colored nodes represent five distinct MR analysis methods. The confidence in these four metabolites and their ratios remains high.

### 3.5 Metabolic pathway analysis

**FIGURE 6:**
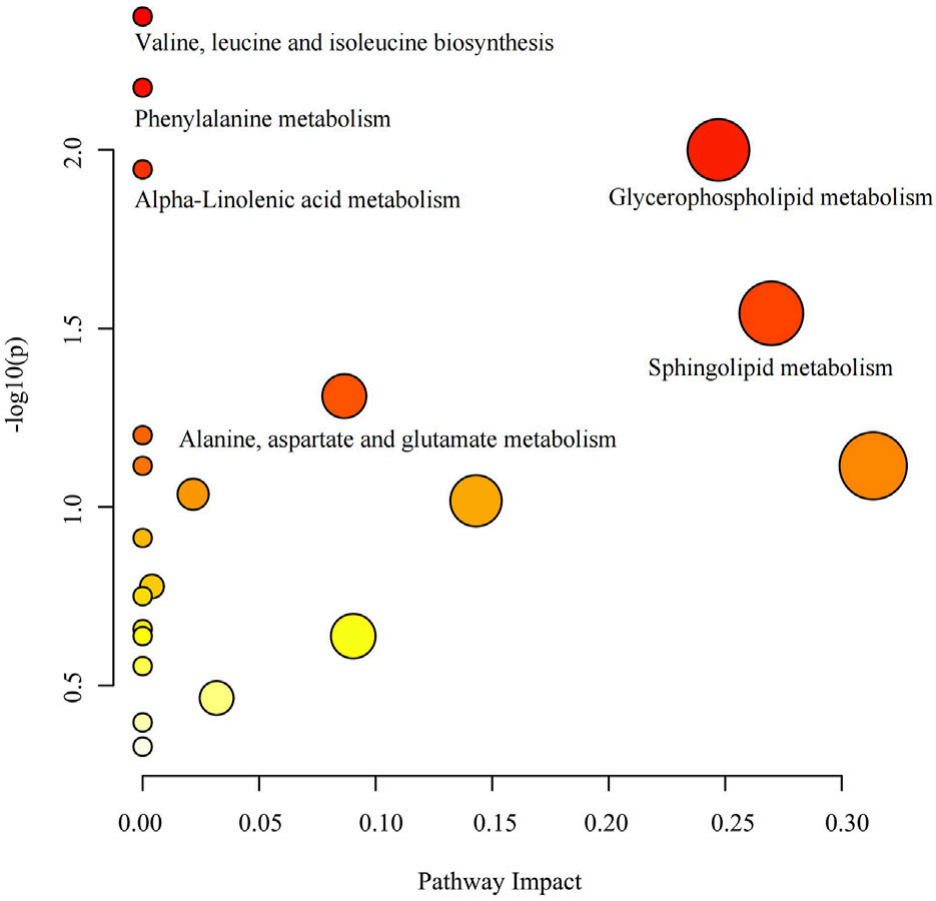
Metabolic pathway analysis bubble diagram.

Pathway analysis of 74 known metabolites with statistically significant (*p* < 0.05) IVW results unveiled six metabolic pathways with considerable significance (*p* < 0.05): Valine, leucine, and isoleucine biosynthesis (*p* = 0.004), Phenylalanine metabolism (*p* = 0.007), Glycerophospholipid metabolism (*p* = 0.010), Alpha-Linolenic acid metabolism (*p* = 0.011), Sphingolipid metabolism (*p* = 0.029), and Alanine, aspartate, and glutamate metabolism (*p* = 0.049) (Supplementary Table S7) (Figure 6). The nodes in the graph depict metabolic pathways, wherein nodes that are higher and darker indicate smaller *p*-values associated with the respective metabolic pathway.

## 4 Discussion

The substantial global burden of high morbidity and mortality rates associated with T2DM has underscored the urgent need for early screening and prevention measures (Collins et al., 2011; Mulnier et al., 2006; Ogurtsova et al., 2022). Achieving high-quality medical care necessitates tailored and precise treatments, crucial for foreseeing individual health indicators and preventing the onset of T2DM(Hampel et al., 2021). Biomarkers serve as pivotal tools in disease management and prevention. Personalized and multidimensional biomarkers significantly contribute to predicting, diagnosing, and prognosing T2DM, offering invaluable insights for drug development, clinical diagnosis, and individualized treatment strategies. The advancement of histological technology facilitates understanding the molecular mechanisms underlying T2DM and evaluating biomarkers, thereby significantly advancing the realm of precision medicine for T2DM.

This study represents an extensive investigation into T2DM, integrating genomics and metabolomics. We utilized GWAS data from two large-scale T2DM cohorts to examine the causal relationship between 1,091 blood metabolites and 309 metabolite ratios with T2DM. Initially, employing IVW, Weighted Median, MR Egger, Weighted Mode, and Simple Mode methods, we identified 185 metabolites and 70 metabolite ratios with at least one method showing significant results (*p* < 0.05). Specifically, the IVW analysis revealed 88 blood metabolites and 37 metabolite ratios displaying a significant causal association with T2DM (*p* < 0.05), encompassing 74 known metabolites. From these 74 metabolites, six metabolic pathways potentially involved in T2DM pathogenesis were identified: Valine, leucine, and isoleucine biosynthesis (*p* = 0.004), Phenylalanine metabolism (*p* = 0.007), Glycerophospholipid metabolism (*p* = 0.010), Alpha-Linolenic acid metabolism (*p* = 0.011), Sphingolipid metabolism (*p* = 0.029), and Alanine, aspartate, and glutamate metabolism (*p* = 0.049). Additionally, through FDR multiple test correction, we identified three known metabolites and one metabolite ratio: 1-linoleoyl-GPE (18:2), 1,2-dilinoleoyl-GPE (18:2/18:2), Mannose, and the Phosphate to Mannose ratio. Subsequently, we validated these findings using another GWAS dataset for T2DM with a substantially larger sample size (Xue et al., 2018), and fortunately, these three known metabolites and one metabolite ratio remained significantly associated in this independent dataset.

The exposure data used in this study originated from Chen’s GWAS investigation (Chen et al., 2023), which stands as the most recent and comprehensive study incorporating metabolites. Consequently, the metabolites under examination are relatively novel, with many of their connections to T2DM yet to be explored. Among our findings, the most significant causal link identified was between 1-linoleoyl-GPE (18:2) and T2DM. Elevated levels of 1-linoleoyl-GPE (18:2) were notably associated with a reduced risk of developing T2DM. However, it is important to note that 1-linoleoyl-GPE (18:2) lacks prior studies exploring its correlation with T2DM. Nevertheless, research has highlighted the significance of linoleoylethanolamide in ameliorating weight gain, dyslipidemia, and inflammation induced by a high-fat diet (Tovar et al., 2023). Considering the established association between obesity, fat accumulation, insulin resistance, and T2DM, it is plausible that 1-linoleoyl-GPE (18:2) may act as a protective factor. Another significant protective metabolite identified in our study is 1,2-dilinoleoyl-GPE (18:2/18:2), yet there is a lack of studies exploring its relevance to T2DM. Jansen’s team (Jansen et al., 2023) observed that 1,2-Dilinoleoyl-sn-glycero-3-phosphocholine enhances adipocyte catabolism and apoptosis through a TNF-α-dependent pathway, thereby alleviating insulin resistance *via* PPARα-mediated inhibition of myocyte inflammation. Furthermore, our study has identified Mannose as a risk factor for the development of T2DM. Mannose, a crucial hexose for glycoprotein synthesis, consistently demonstrates a significant association with elevated blood glucose levels and T2DM development in prospective studies (Carter et al., 2016; Floegel et al., 2013; Menni et al., 2013; Sone et al., 2003). This relationship might elucidate the Phosphate to Mannose ratio, where Mannose, as the denominator, potentially serves as a protective factor against T2DM. Among the results obtained from metabolic pathway analysis, the Valine, leucine, and isoleucine biosynthesis pathways exhibited the highest significance (*p* = 0.004). Valine, leucine, and isoleucine within this metabolic pathway act as vital nutritional signals influencing protein synthesis, glucose regulation, and anti-obesity mechanisms (Nie et al., 2018). Specifically, isoleucine stimulates glucose uptake in skeletal muscle, thereby preventing spikes in plasma glucose concentrations, while also exhibiting a preventive effect against visceral obesity and hyperinsulinemia (Doi et al., 2005; Nishimura et al., 2010).

Our study exhibits several strengths. Firstly, we leveraged the most advanced and comprehensive GWAS data encompassing 1,091 blood metabolites and 309 metabolite ratios. Consequently, our study stands as the most cutting-edge exploration of causal associations between metabolites and T2DM to date. Moreover, we implemented a rigorous MR study methodology to mitigate confounding factors commonly present in observational studies. To ensure the robustness of our findings, we conducted a series of sensitivity analyses, enhancing the reliability of our results. Additionally, we validated our findings using an alternate, larger sample set from a separate GWAS dataset for T2DM, obtaining consistently aligned results with our original study. Lastly, our utilization of metabolic pathway analyses offers insights into the metabolic mechanisms associated with the onset of T2DM, providing valuable reference points for further investigation.

Nevertheless, this study bears certain limitations. Initially, our inclusion covered a relatively restricted subset of the 1,400 exposed SNPs, thereby necessitating a more permissive threshold during the screening of instrumental variables for MR analysis, akin to other studies of a similar nature. Additionally, a limited number of metabolites were inevitably omitted from pathway analysis due to lacking nomenclature or annotations in the metabolic pathway database. To address these limitations, future investigations should prioritize further experimentation on less explored metabolites, offering a more comprehensive understanding of their association with T2DM. Moreover, expanding the sample size of the original dataset and conducting high-quality randomized controlled trials alongside fundamental studies will be pivotal for validating our findings in subsequent research.

## 5 Conclusion

To summarize, this MR analysis unveiled 88 blood metabolites and 37 metabolite ratios exhibiting significant causal links to T2DM. Furthermore, through FDR validation, three established metabolites and one metabolite ratio emerged as displaying the most robust causal association with T2DM. Additionally, our investigation identified six metabolic pathways potentially linked to T2DM development. These discerned serum metabolites establish a foundation for early screening, preventive strategies, and treatment protocols for T2DM, while also guiding the blueprint for future clinical studies. The amalgamation of genomics and metabolomics in MR analysis serves as a pivotal pathway for delving into the etiology and pathogenesis of T2DM.

## Data Availability

The datasets presented in this study can be found in online repositories. The names of the repository/repositories and accession number(s) can be found in the article/Supplementary Material.
