# Supplementary material for: Genetically predicted 1091 blood metabolites and 309 metabolite ratios in relation to risk of type 2 diabetes: a Mendelian randomization study
Source: Front Genet. 2024 Jul 10;15:1356696. doi: 10.3389/fgene.2024.1356696 (PMC11266066; doi:10.3389/fgene.2024.1356696)
Supplement: Supplementary file 1 [file Table1.DOCX]

Supplementary Material

# Supplementary Figures


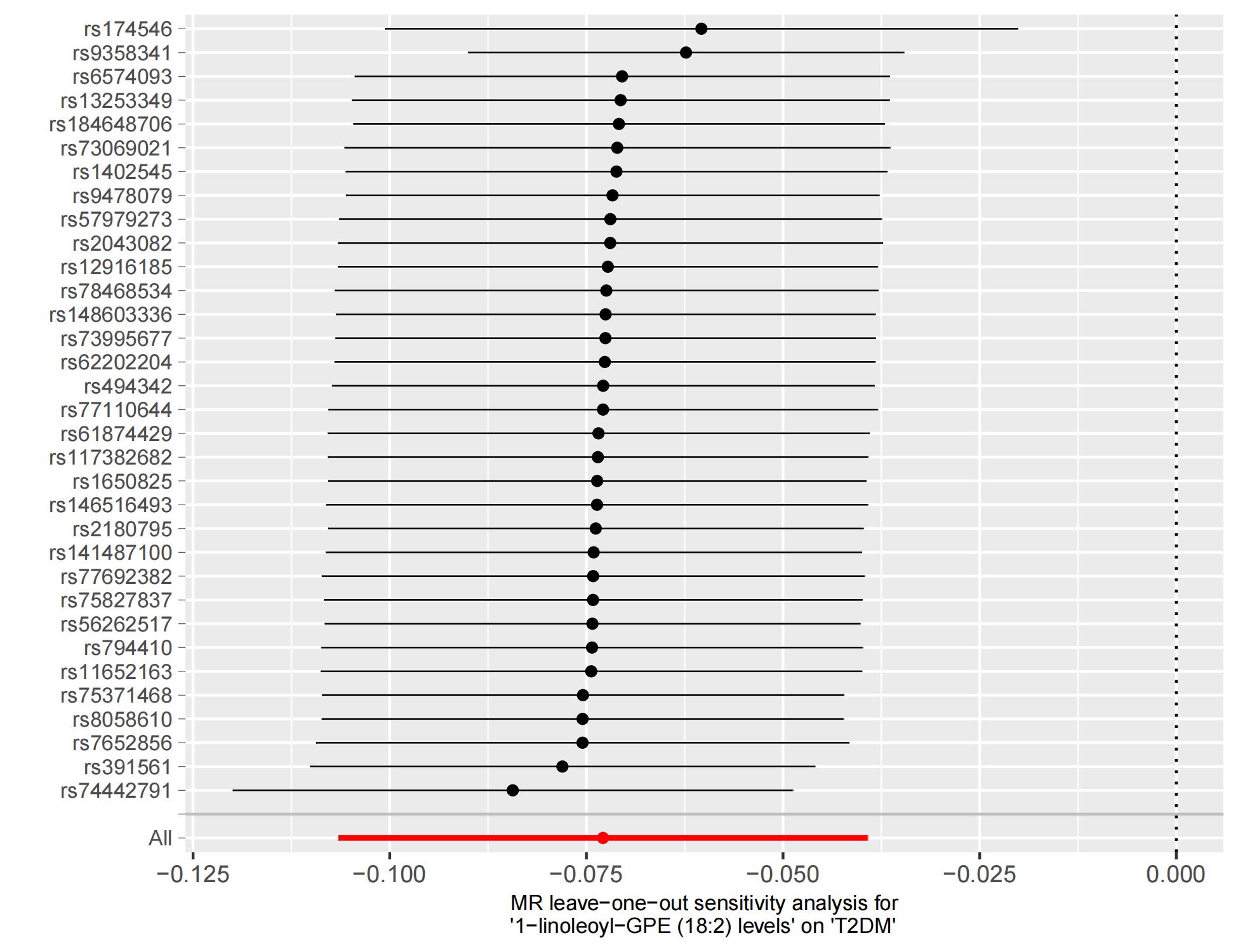


**Supplementary Figure 1.** Leave-one-out analysis of causal associations between 1-linoleoyl-GPE (18:2) and T2DM.


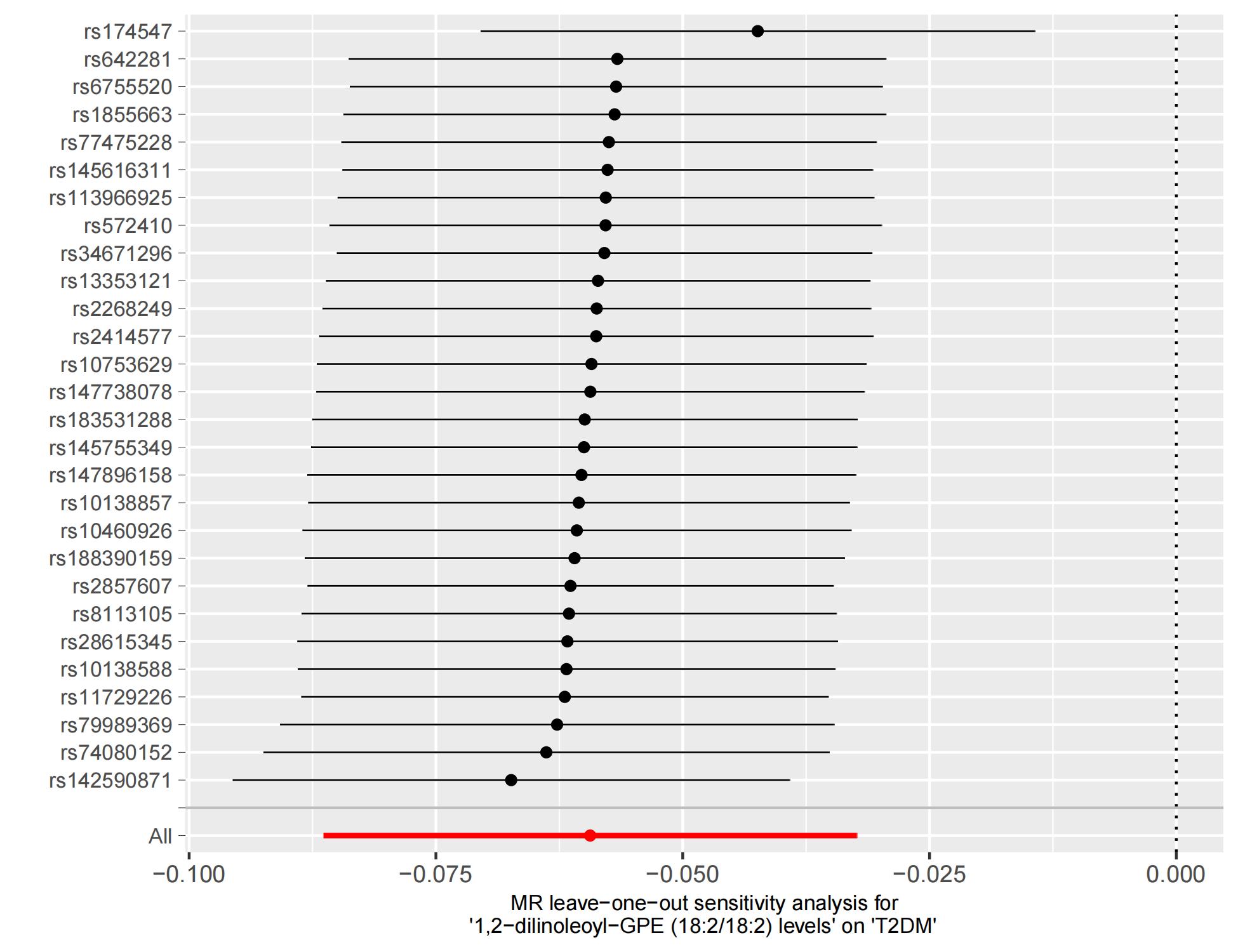


**Supplementary Figure 2.** Leave-one-out analysis of causal associations between 1,2-dilinoleoyl-GPE (18:2/18:2) and T2DM


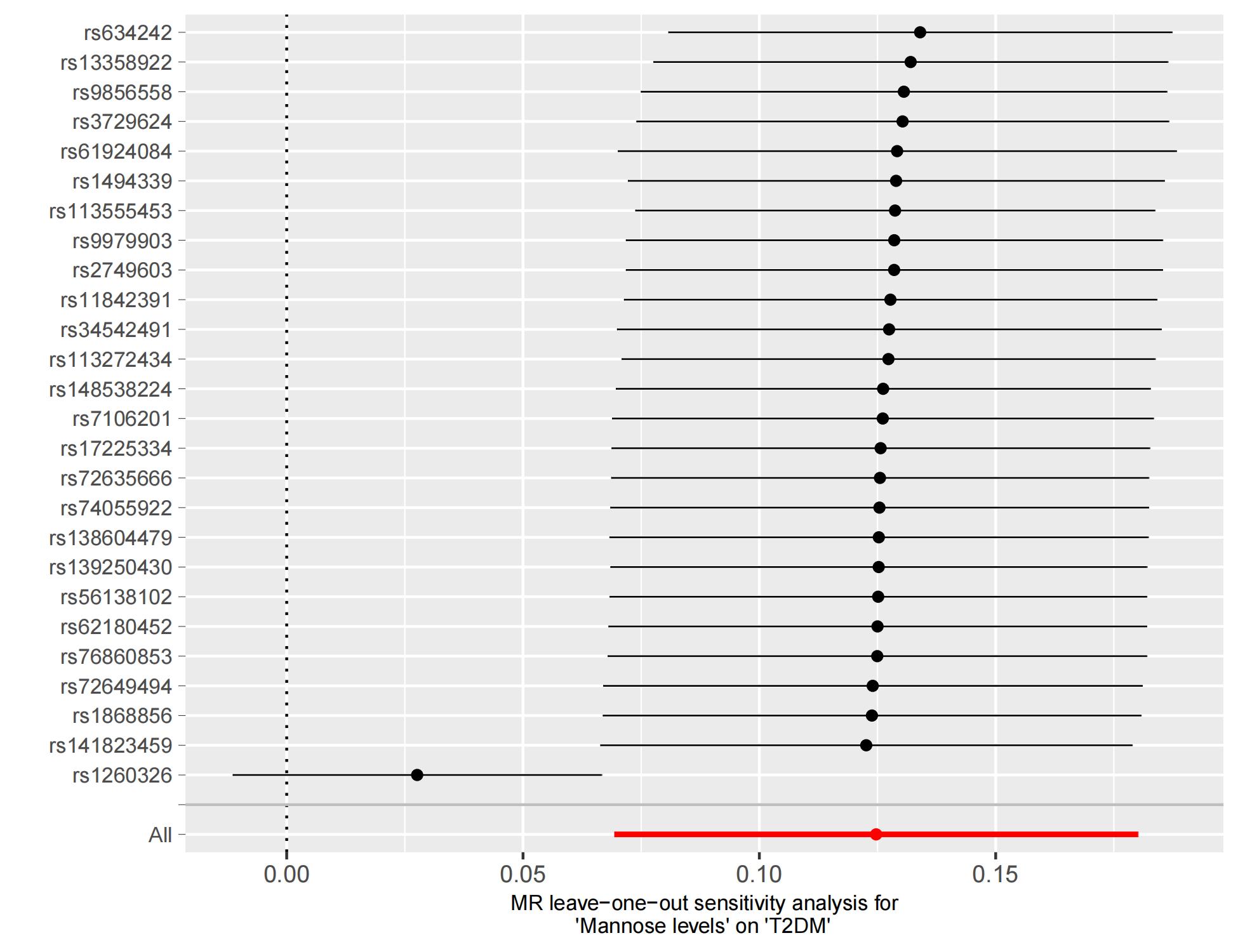


**Supplementary Figure 3.** Leave-one-out analysis of causal associations between Mannose and T2DM


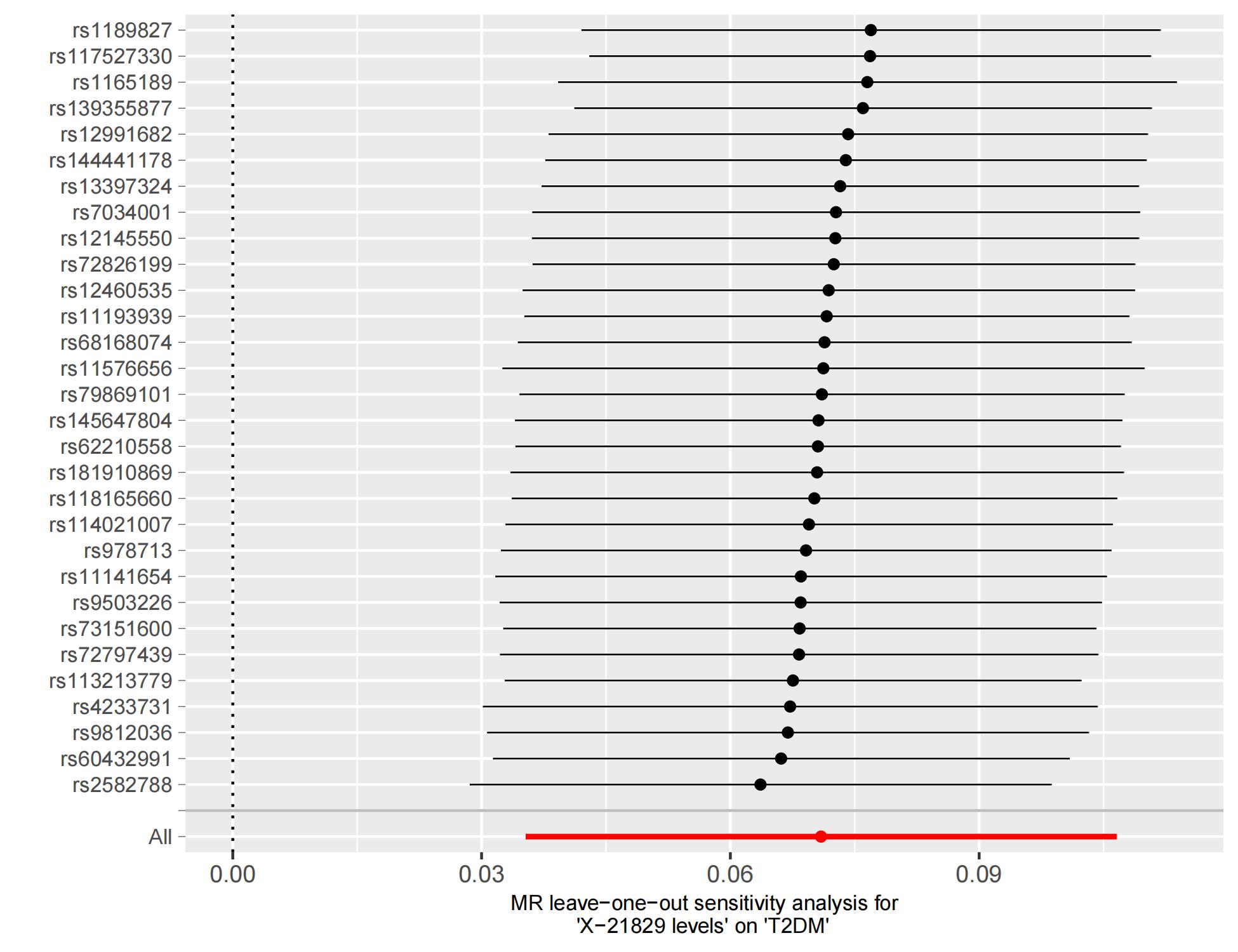


Supplementary Figure 4. Leave-one-out analysis of causal associations between X-218:29 and T2DM


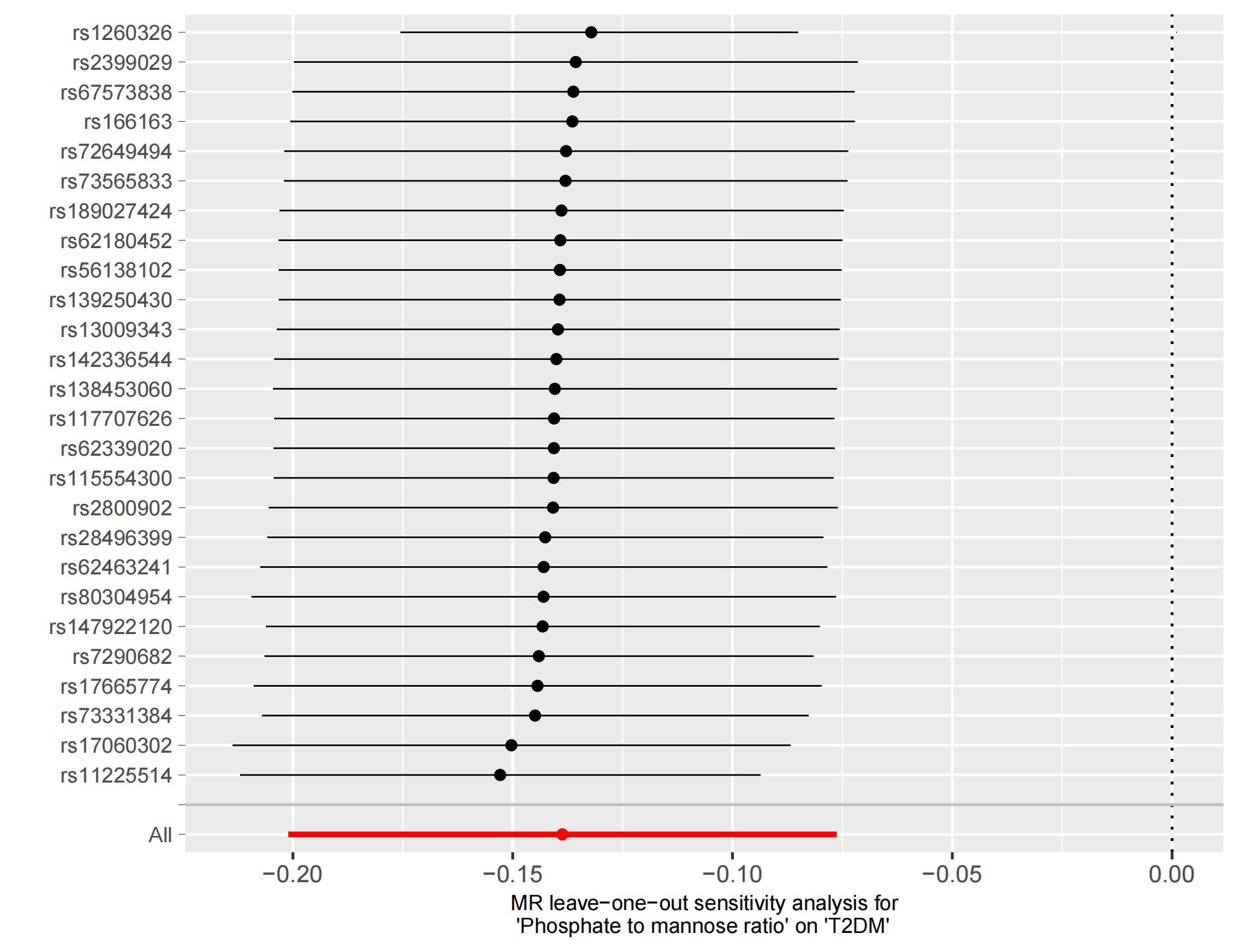


**Supplementary Figure 5.** Leave-one-out analysis of causal associations between Phosphate to mannose ratio and T2DM


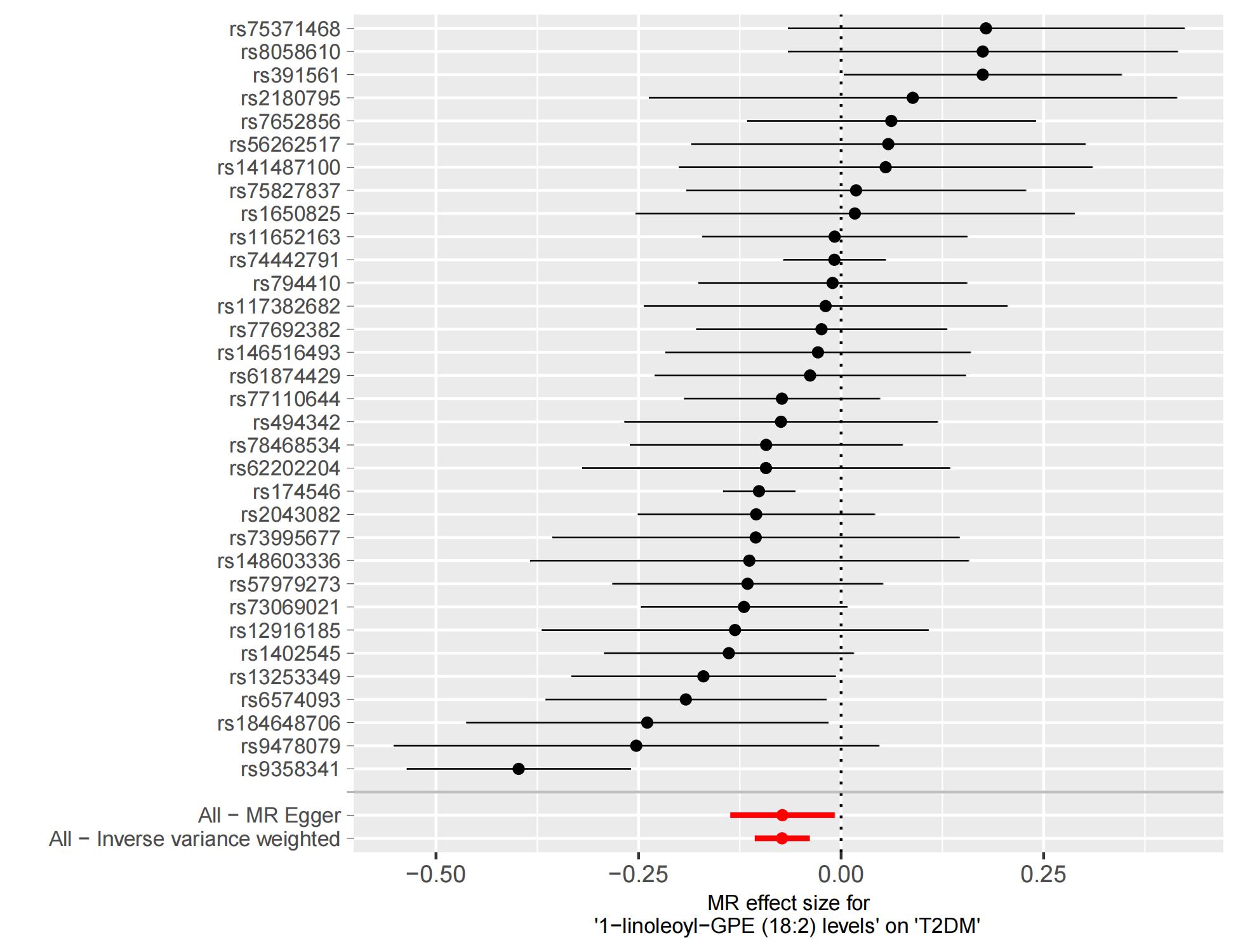


**Supplementary Figure 6.** Individual SNP analysis of causal associations between 1-linoleoyl-GPE (18:2) and T2DM


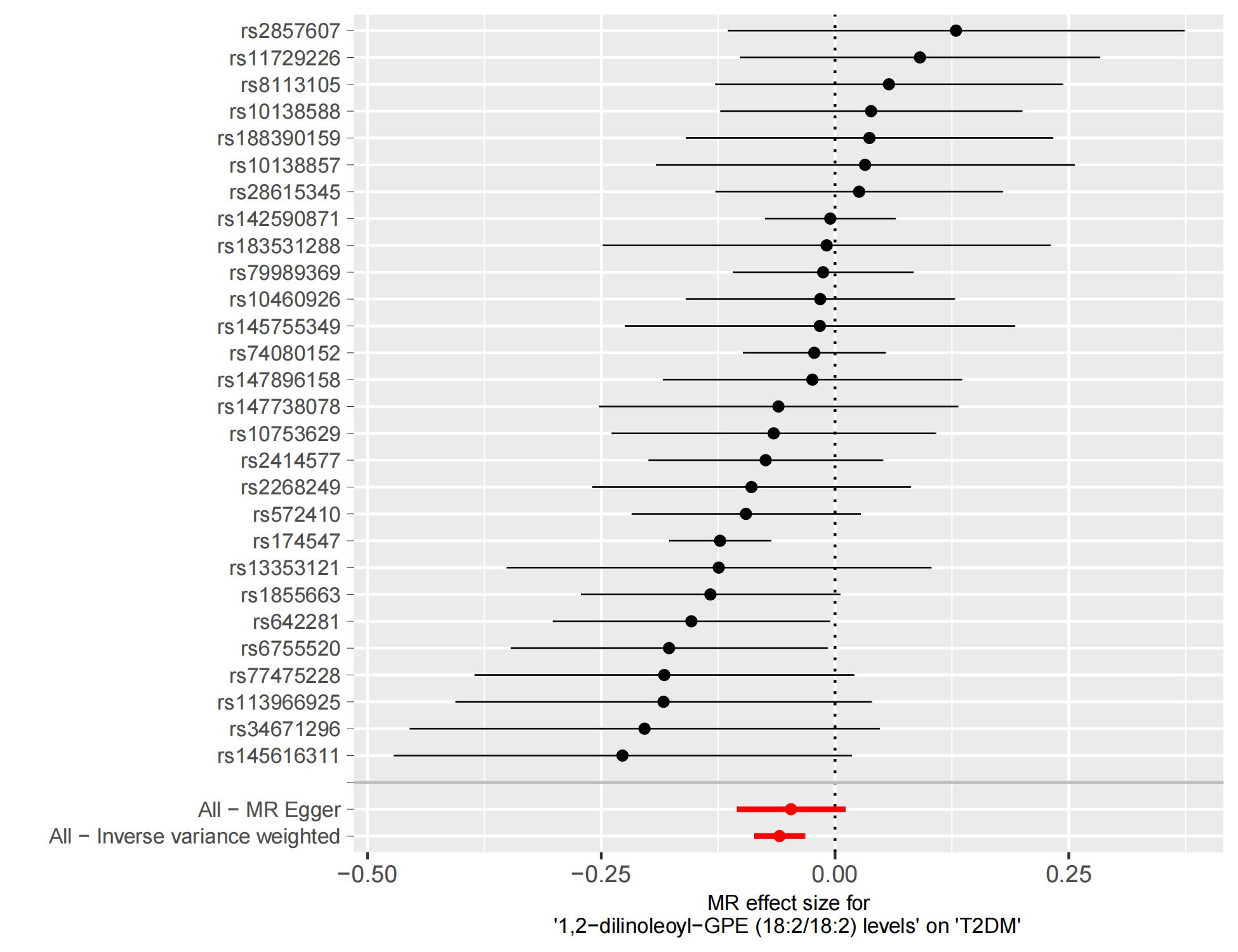


Supplementary Figure 7. Individual SNP analysis of causal associations between 1,2-dilinoleoyl-GPE (18:2/18:2) and T2DM


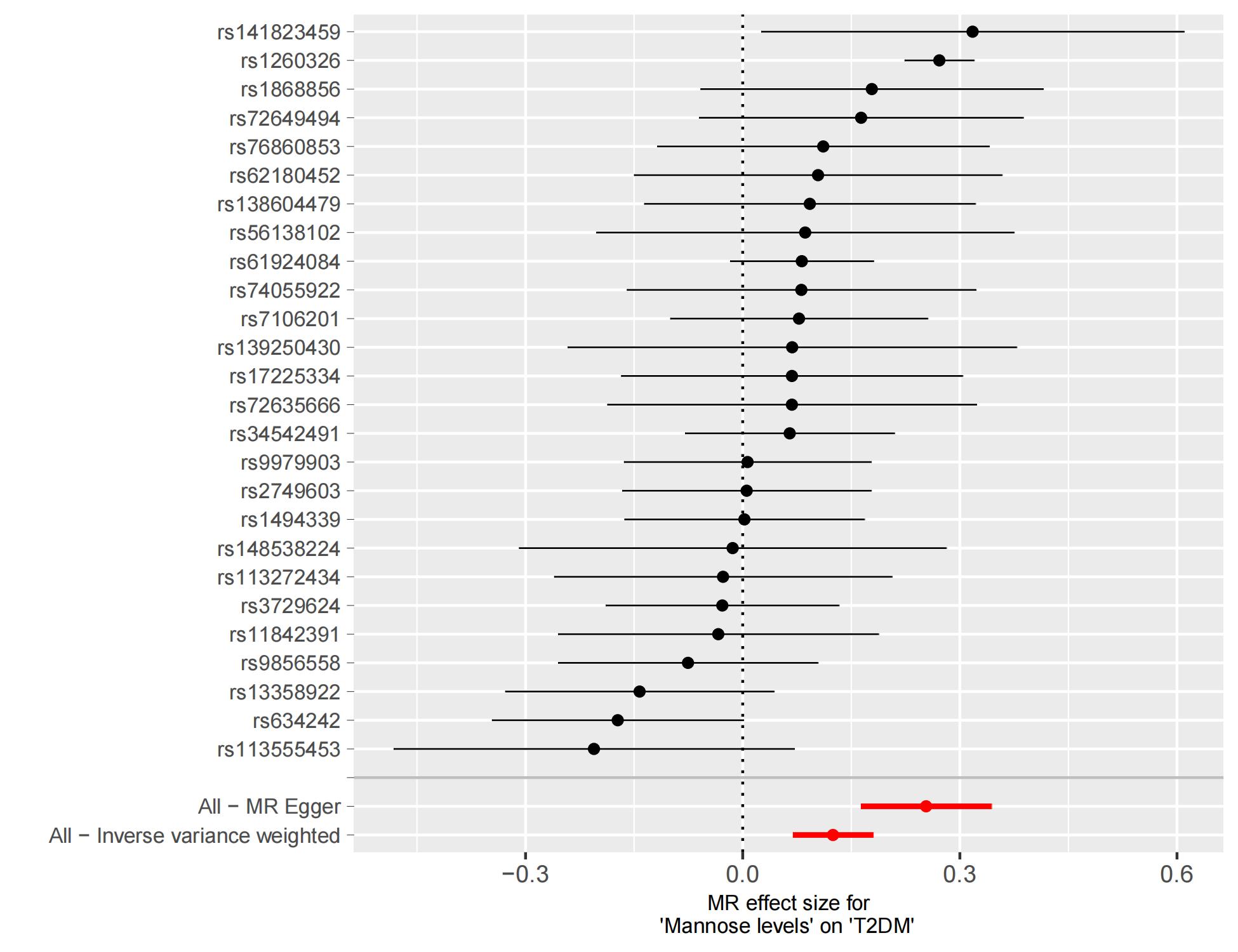


**Supplementary Figure 8.** Individual SNP analysis of causal associations between Mannose and T2DM


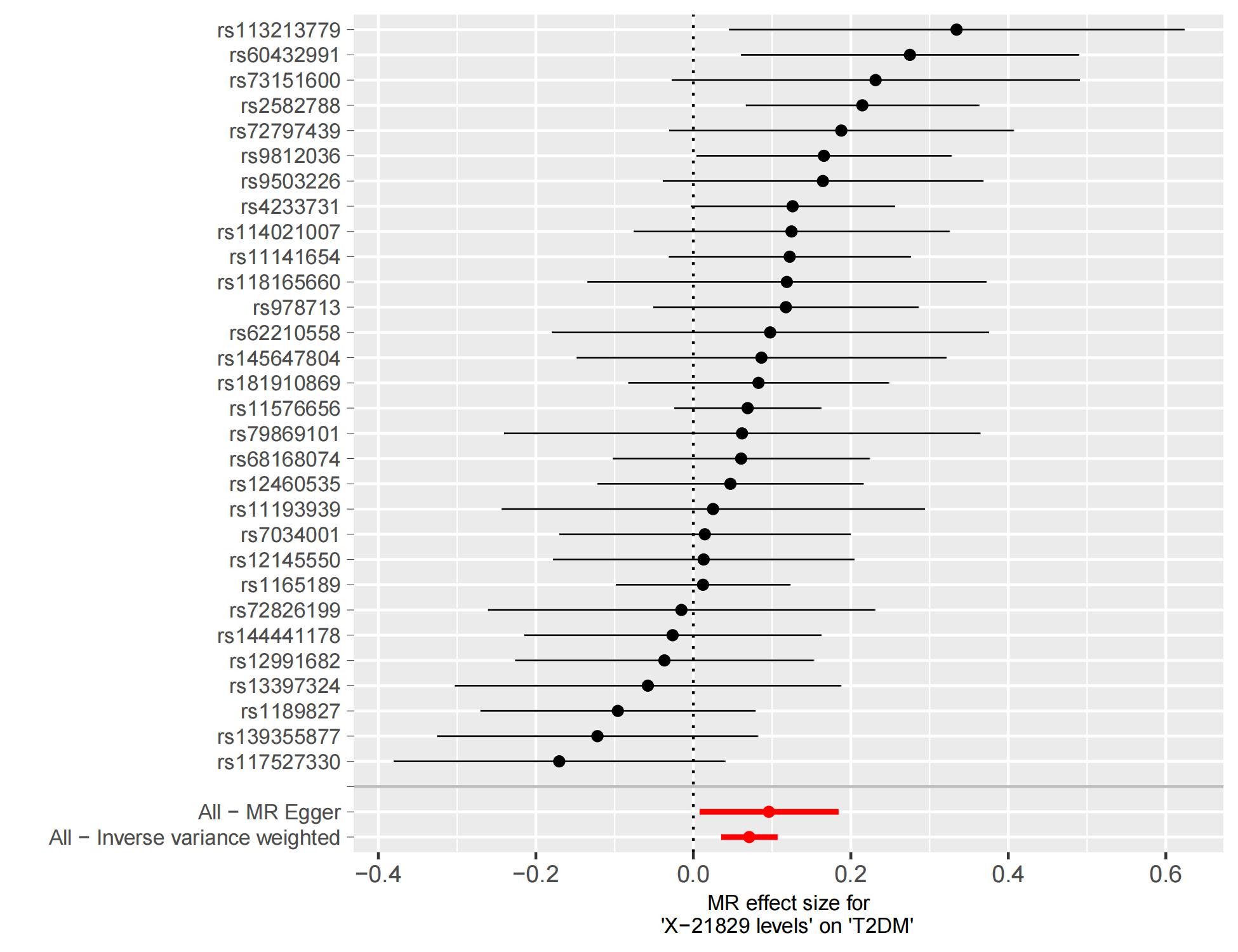


**Supplementary Figure 9.** Individual SNP analysis of causal associations between X-218:29 and T2DM


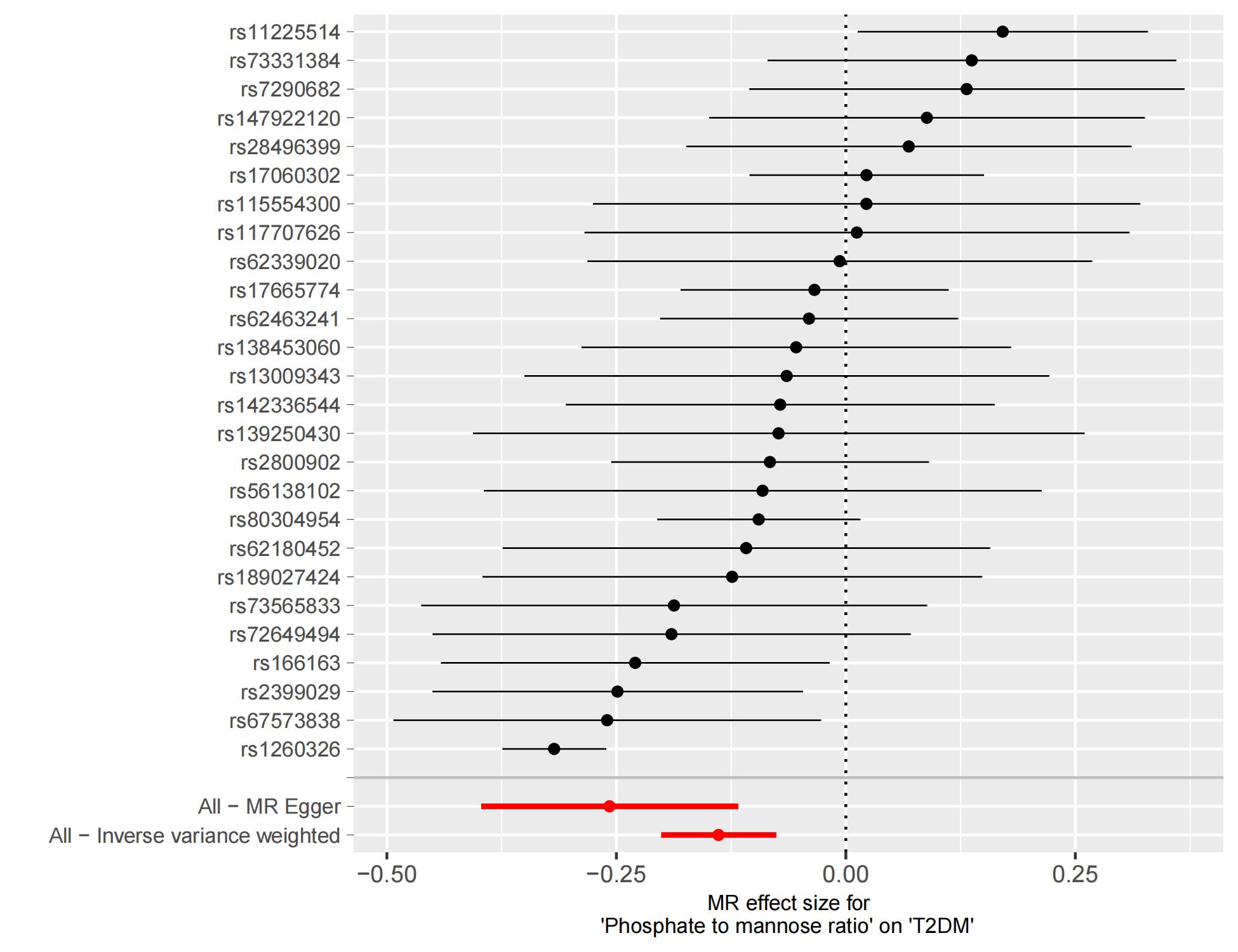


**Supplementary Figure 10.** Individual SNP analysis of causal associations between Phosphate to mannose ratio and T2DM


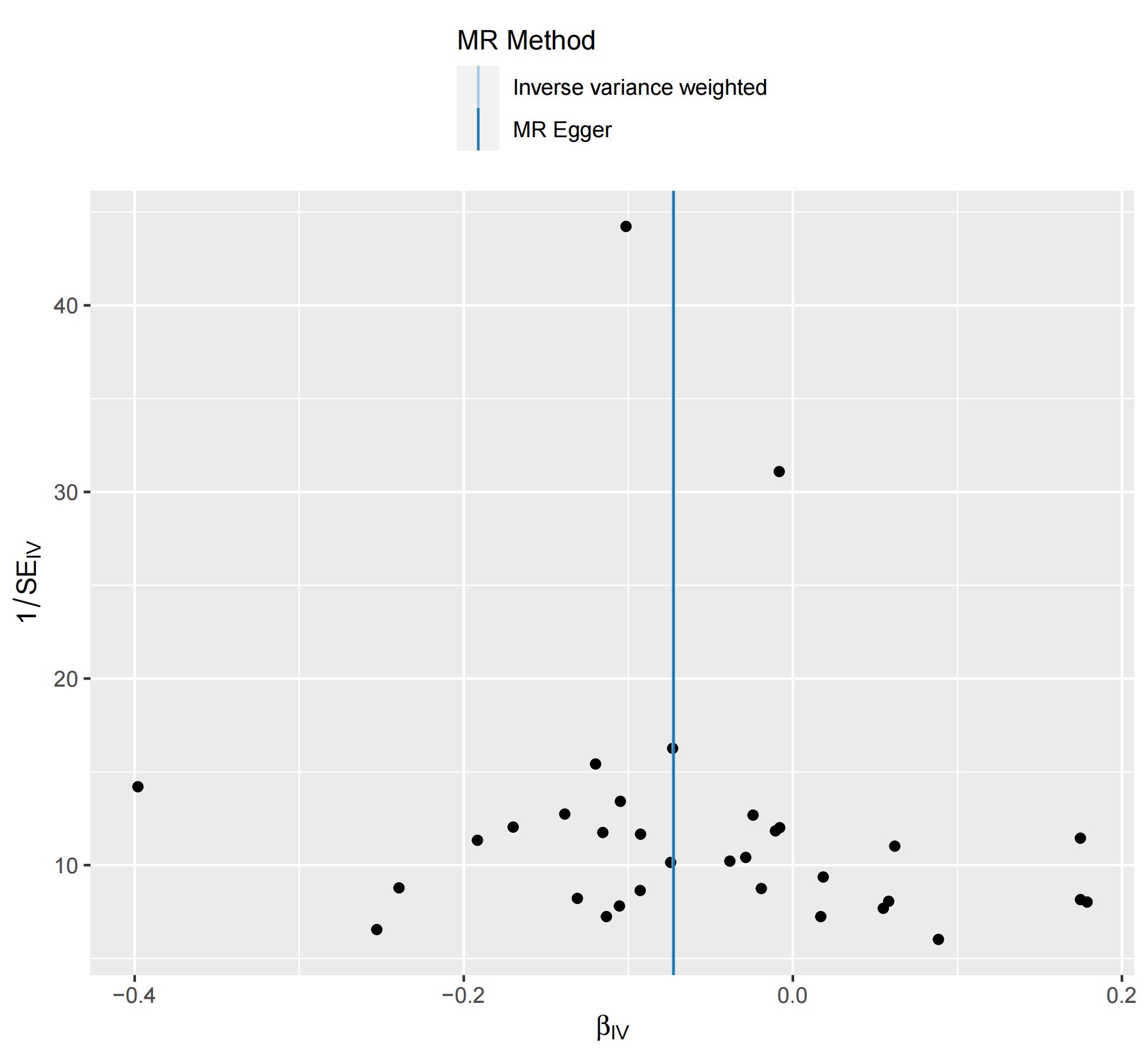


**Supplementary Figure 11.** Funnel plot of causal associations between 1-linoleoyl-GPE (18:2) and T2DM


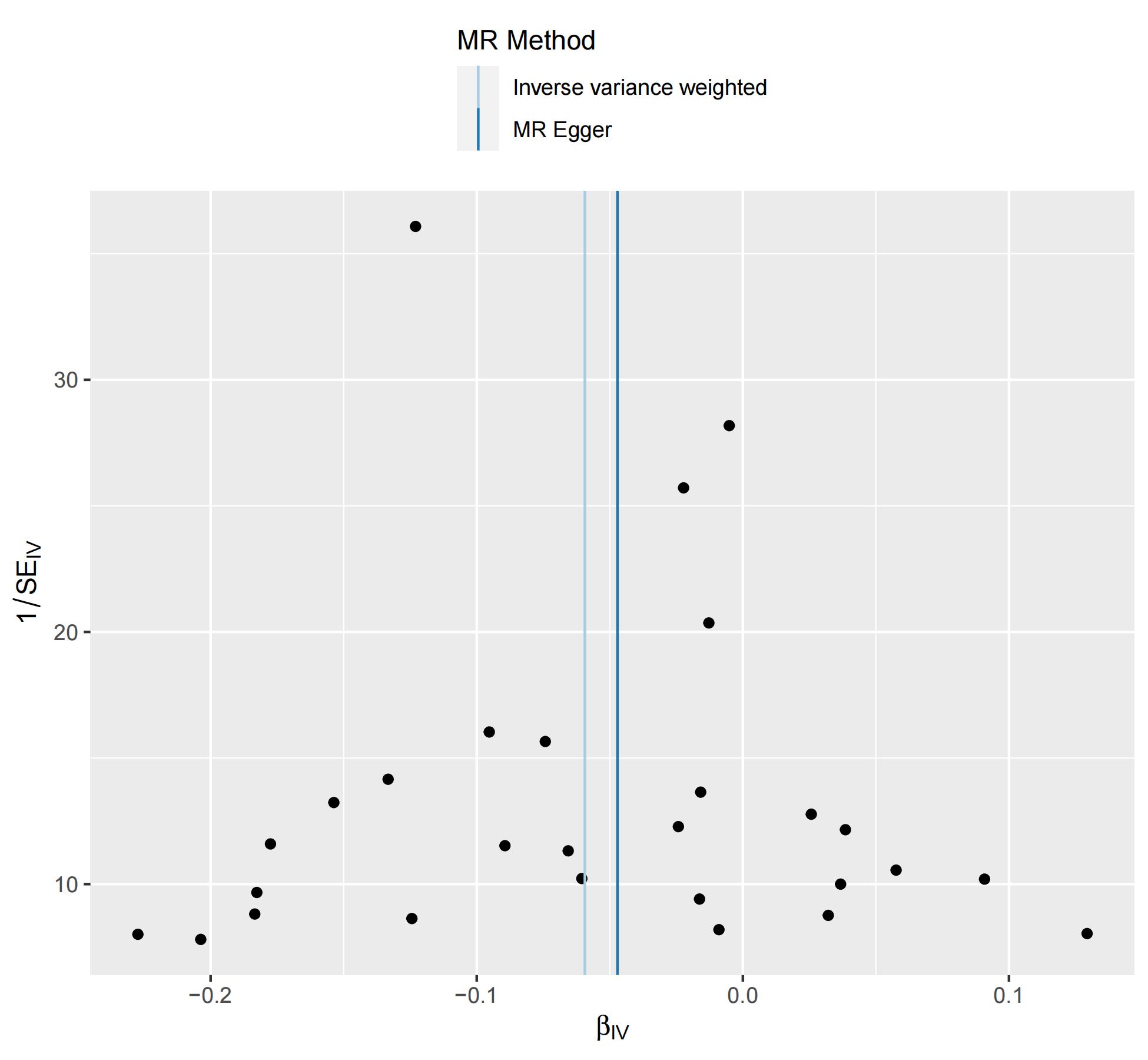


**Supplementary Figure 12.** Funnel plot of causal associations between 1,2-dilinoleoyl-GPE (18:2/18:2) and T2DM


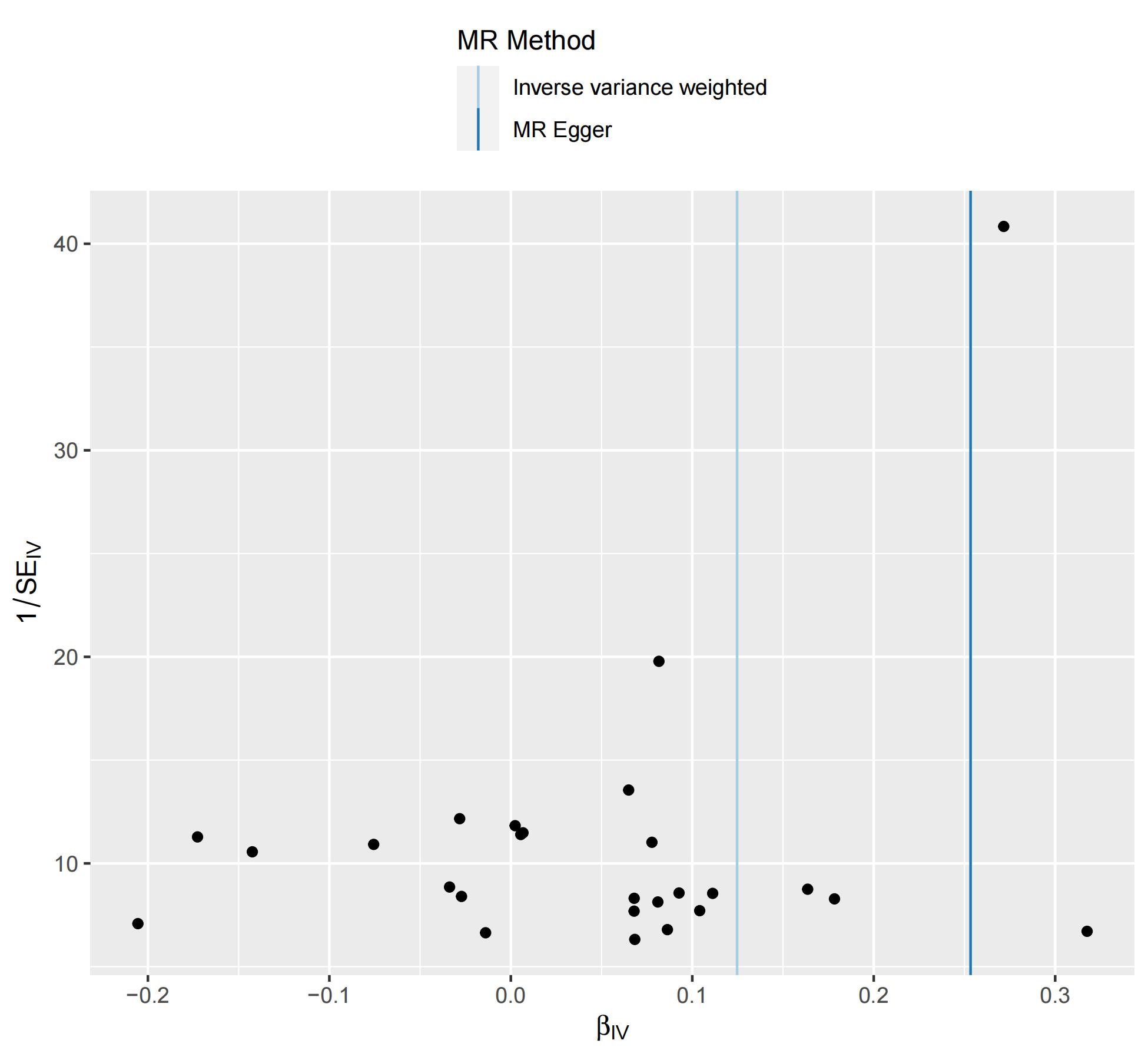


**Supplementary Figure 13.** Funnel plot of causal associations between Mannose and T2DM


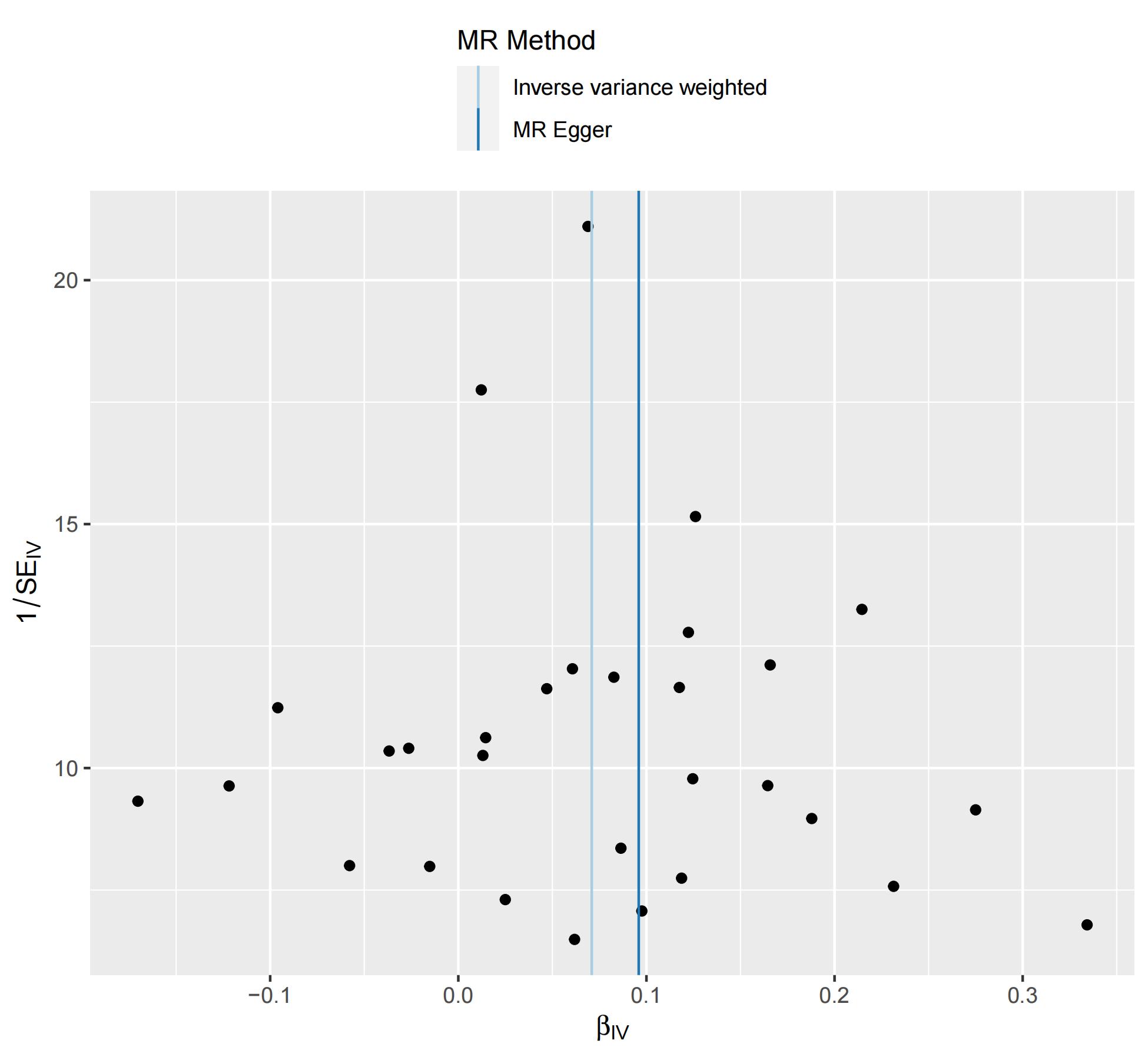


**Supplementary Figure 14.** Funnel plot of causal associations between X-218:29 and T2DM

**
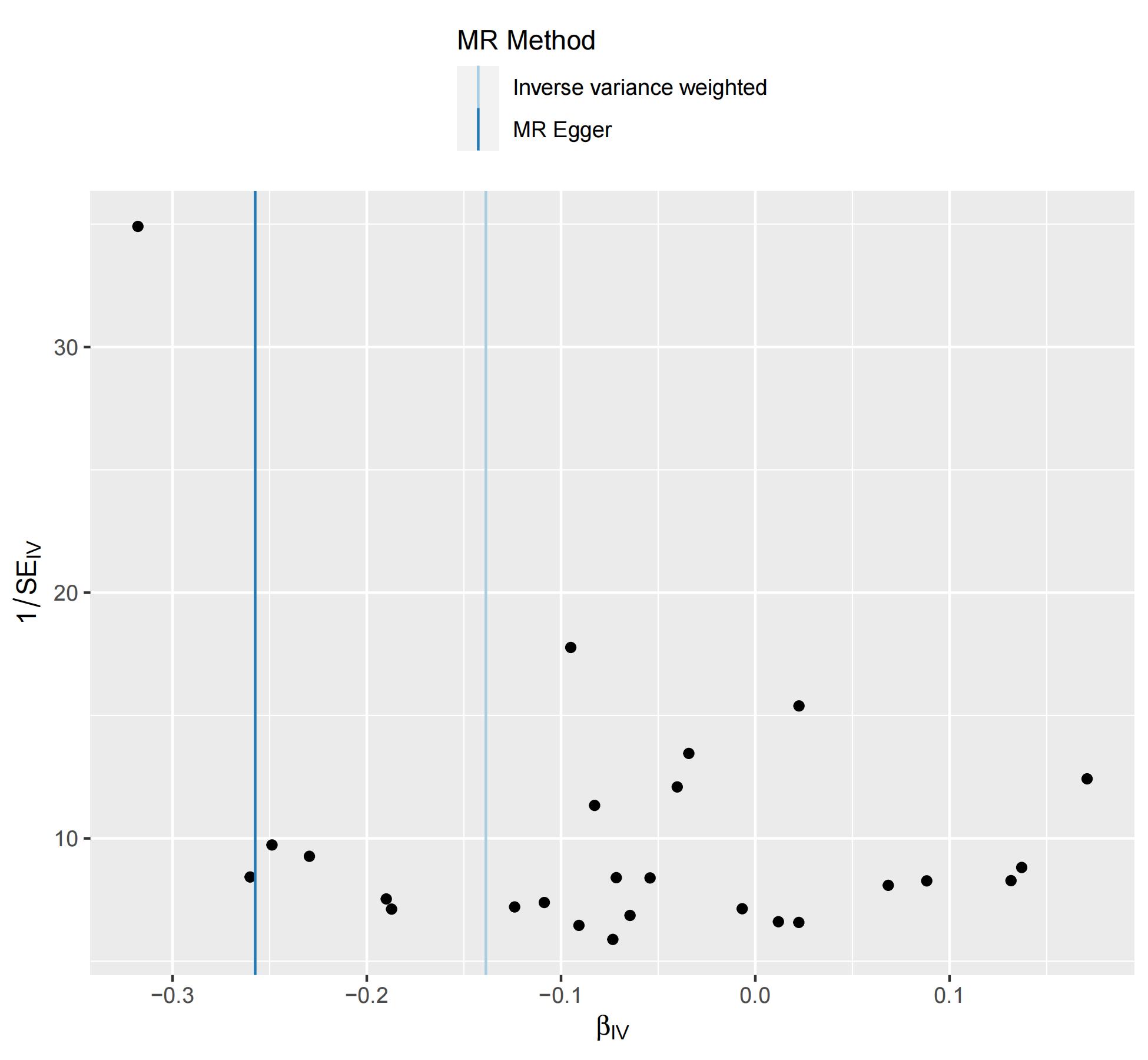
**

**Supplementary Figure 15.** Funnel plot of causal associations between Phosphate to mannose ratio and T2DM
